# Current Trends of Nanotechnology in Orthodontics

**DOI:** 10.7759/cureus.69035

**Published:** 2024-09-09

**Authors:** Pinaki Roy, Poulomi Roy

**Affiliations:** 1 Orthodontics and Dentofacial Orthopedics, Burdwan Dental College and Hospital, Bardhaman, IND

**Keywords:** nanodentistry, nanomaterials, nanoparticles, nanorobots, nanotechnology, orthodontics

## Abstract

The branch of science known as nanotechnology studies the manipulation of matter at the nanoscale. The field of nanotechnology is expanding daily and has shown great promise in all areas of medicine, including dentistry. Because of its many uses, including nanocoatings in brackets and archwires, orthodontic bonding, antimicrobial qualities, and atomic force microscopy, it has also become more significant in the field of orthodontics. Some of its potential uses in the future include shape-memory polymers, gene therapy-induced stimulation of mandibular growth, accelerating orthodontic movement, and biomechanical sensors. The capacity of nanotechnology to improve material qualities, particularly antimicrobial properties, has led to its increased prominence in recent years. Many areas of orthodontics can benefit from the application of nanotechnology. This paper focuses on the impact of nanomaterials on orthodontic appliances and treatment.

## Introduction and background

**Figure 1:**
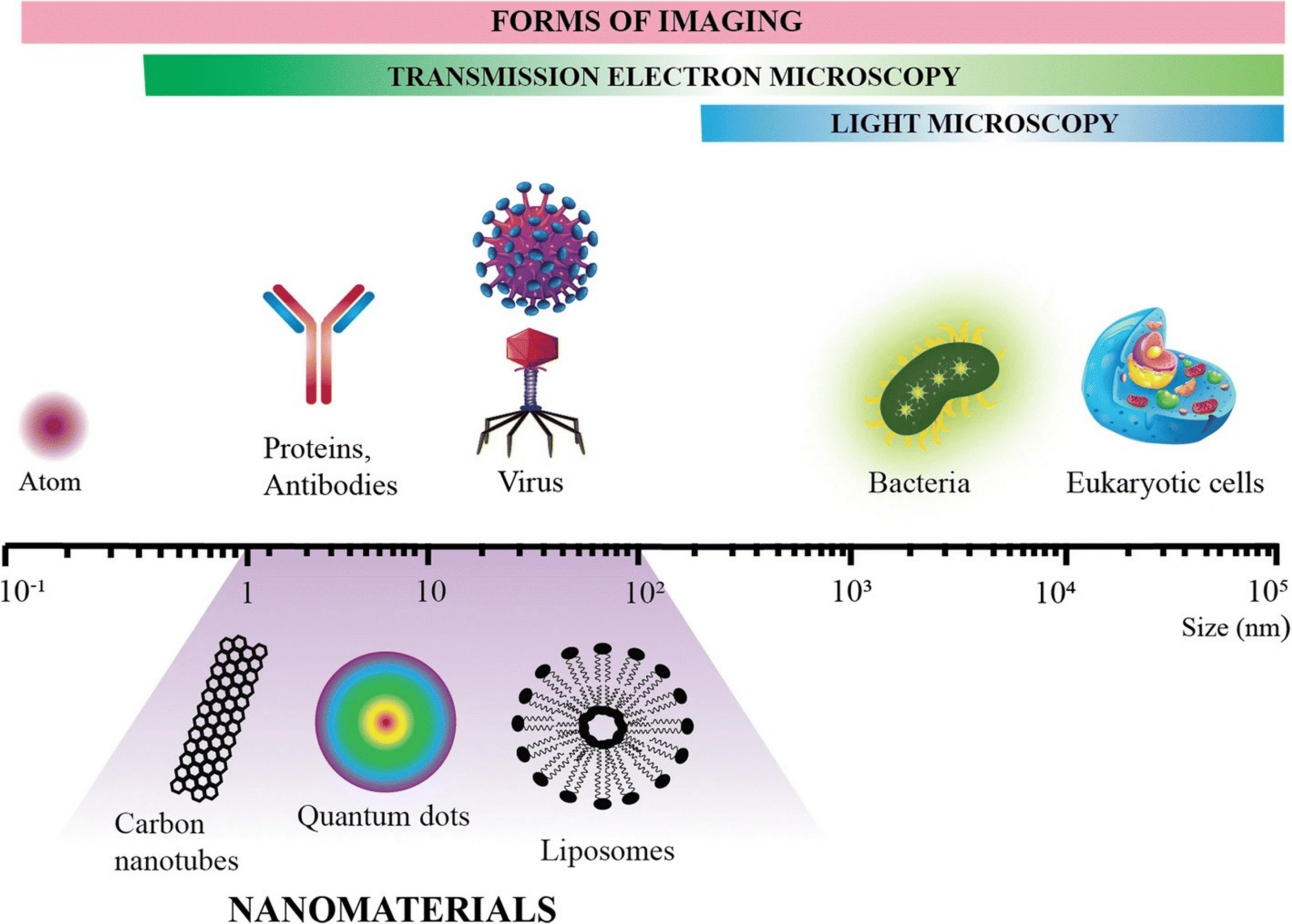
Relative size comparison of nanomaterials and microbiological and other biological entities. Bodies visible by light and transmission electron microscopy are indicated, and a scale bar denotes the size range of the respective biological entities and nanomaterials (1-100 nm) nm: nanometer Reference: [1]

The word "nano" comes from the Greek word for "dwarf." The science of modifying matter, expressed in billionths of meters or nanometers, roughly the size of two or three atoms, is known as nanotechnology. Its action at a scale of one billionth of a meter, or one ten thousandth of the width of a human hair, is what primarily sets it apart (Figure 1). To put it simply, it's atomic or molecular engineering.

It was Richard Zsigmondy, the 1925 Chemistry Nobel laureate, who first put forth the idea of a "nanometer." He was the first to use a microscope to measure the size of particles like gold colloids and is credited with coining the term "nanometer" for the precise description of particle size.

The concept and inception of nanotechnology are attributed to American physicist and Nobel laureate Richard Feynman in 1959. He presented a paper titled "There is Plenty of Room at the Bottom" at the annual meeting of the American Physical Society held at the California Institute of Technology on December 29, 1959 [2].

Although Taniguchi [3] coined the term "nanotechnology" in 1974, it wasn't well-known until Eric Drexler made it popular in his book "Engines of Creation." Since the mid-1990s, it has been a part of accepted logic with potential applications in medicine and dentistry.

Early in the 2000s, nanoparticles like titanium dioxide, zinc oxide, and silver nanoparticles were used in commercial products utilizing nanotechnology. The addition of nanoingredients alters the materials' mass properties. There are two possible explanations for why the material's properties changed at the nanoscale: Firstly, the nanomaterials act in a more artificially responsive way, influencing their quality or electrical properties, due to their larger surface area when compared to a similar mass of material delivered in a bigger structure. Secondly, the optical, electrical, and attractive conduct of materials are affected by quantum effects, which demonstrate the more dominant behavior of the challenge at the nanoscale, particularly at the lower end. One measurement, two measurements, or all three measurements can be used to create materials that are nanoscale.

**Figure 2:**
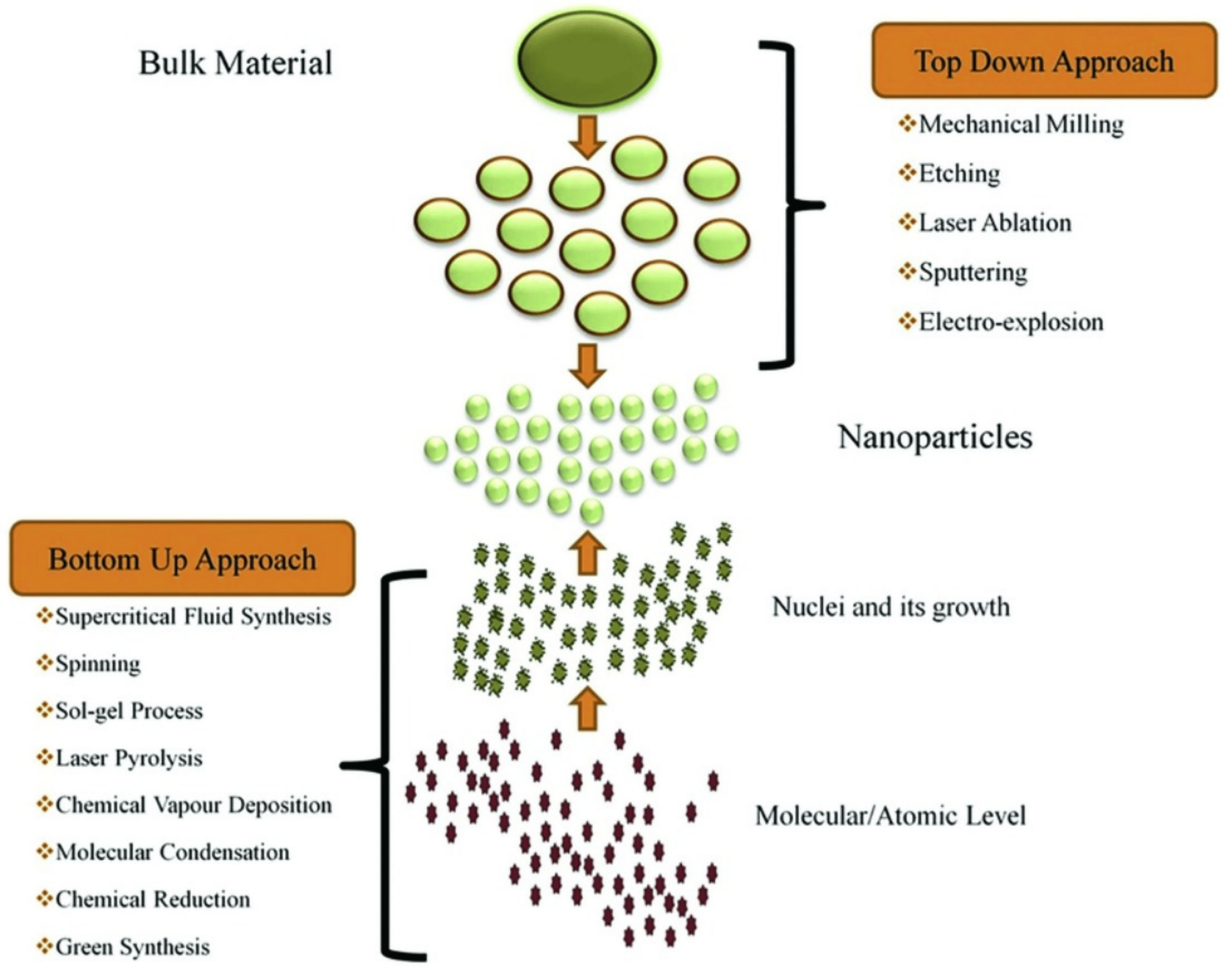
The synthesis of nanomaterials via top-down and bottom-up approaches Reference: [5]

There are two methods of delivering nanomaterial: the top-down approach and the bottom-up approach (Figure 2) [4]. The top-down approach starts with a mass material and uses mechanical, chemical, or other forms of energy to break it into smaller pieces. In contrast, in the bottom-up approach, nanostructures are assembled starting at the bottom, atom-by-atom or molecule-by-molecule, through the controlled manipulation of atom and molecular self-assembly using physical and chemical techniques in the nanoscale range of 1-100 nm.

## Review

Current scope of nanotechnology in orthodontics

Nanotechnology is an underexplored field in its application in orthodontics. Given the biological, technological, and ethical difficulties involved, the elements and concepts pertaining to nanotechnology and orthodontics are difficult to put into practice [6]. The necessary research for the applications of nanotechnology in orthodontic dentistry is still ongoing. Some applications have been tested and are currently being utilized in various sectors, such as to control oral biofilm and as an antimicrobial agent, nano-adhesives for orthodontic bonding, nanocoating in archwires, brackets, bands, and mini-screws, and nanoparticles in power chains and elastic ligatures.

Nanocoatings in Archwires and Brackets

Reducing the frictional forces between the brackets and orthodontic wire may shorten the treatment time by increasing the desired tooth movement. In recent years, nanoparticles have been incorporated into dry lubricants. Without the use of a liquid medium, solid-phase materials known as "dry lubricants" can lower friction between two surfaces that are sliding against one another.

The most effective nanomaterials for accomplishing this objective of lowering friction are thought to be W2 (tungsten disulfide) and MoS2 (molybdenum disulfide) [7]. Three wires composed of titanium molybdenum alloy, nickel-titanium alloy, and stainless steel (SS) were coated with smooth, uniform nanoparticles known as nanoceramics in a study [8]. It was observed that the application of the coating improved the surface topology, which led to a decrease in friction-related issues.

For orthodontic SS wires, inorganic fullerene-like nanoparticles of tungsten sulfide (IF-WS2), which are strong dry lubricants, have been employed as self-lubricating coatings. Redlich et al. [9] inserted SS wires into electroless solutions of nickel-phosphorus (Ni-P) and IF-WS2 to coat the wire with Ni-P electroless film impregnated with inorganic fullerene-like nanoparticles of IF-WS2. An Instron machine was used to perform friction tests that simulated the functioning of an archwire on both coated and uncoated wire. The coated wires' scanning electron microscopy and energy-dispersive X-ray spectroscopy (SEM/EDS) analysis demonstrated a clear impregnation of IF-WS2 nanoparticles in the Ni-P matrix. Up to 54% less friction force was recorded on the coated wire.

In order to produce newer orthodontic brackets, UC3M created a novel material in 2012 that included hard alumina nanoparticles embedded in polysulfone. This material has demonstrated enhanced strength, decreased mechanical and friction resistance, and biocompatibility [10].

**Figure 3:**
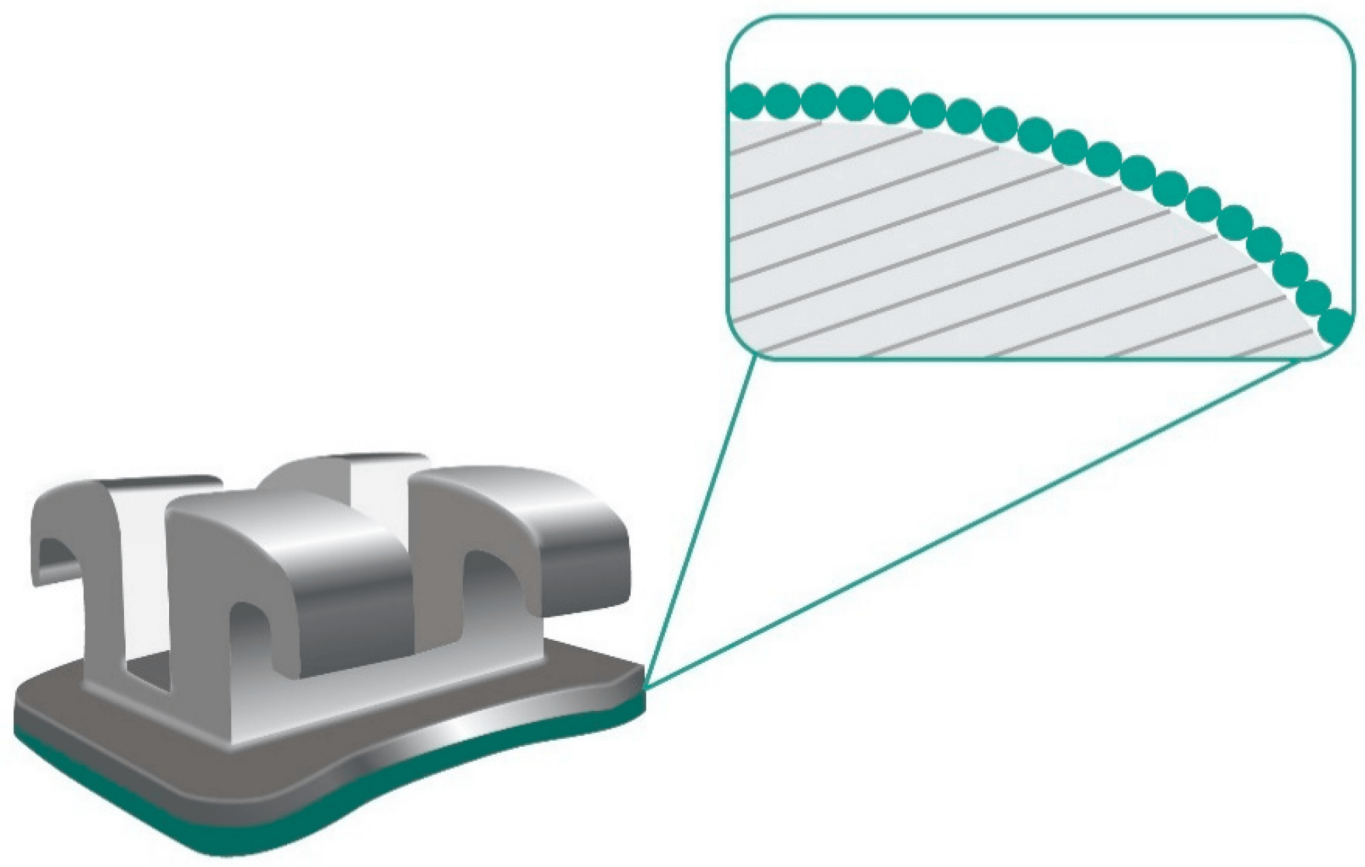
Orthodontic bracket covered by a nanosized film Reference: [12]

Research employing nanocoated brackets (Figure 3) with a thin layer of nitrogen (N2)-doped titanium oxide (TiO2) nanoparticles has demonstrated an antimicrobial effect against oral bacteria. TiO2 exhibits catalytic activity inducing the production of hydroxyl radicals, superoxide ions, peroxyl radicals, and H2O2 due to nitrogen doping and modifications [11]. These radicals have the ability to undergo a sequence of oxidation reactions with biological molecules, causing damage to their biological structure and displaying antimicrobial activity. Antimicrobial activity against *Streptococcus mutans*, *Lactobacillus acidophilus*, *Actinomyces viscosus*, and *Candida albicans* was found to be 95%, 91%, 69%, and 99%, respectively. During orthodontic treatment, gingivitis and enamel demineralization can be avoided with decreased antimicrobial activity.

Nanoparticles in Adhesives

Compared to bracket materials, orthodontic adhesives have a greater ability to hold cariogenic streptococci [12]. Brackets and bands are frequently secured to the tooth surface using composite and glass ionomer cement (GIC) as an adhesive. In orthodontics, nanomaterials can be used as adhesives to increase mechanical strength or lower the chance of enamel damage [13]. Oral streptococci were shown to be resistant to bacteria in resin composites with fillers implanted with silver ions [14]. When compared to conventional adhesives, the addition of silver nanoparticles reduces the adhesion of cariogenic streptococci to orthodontic adhesive without sacrificing physical properties (shear bond strength) [12].

The shear bond strength of brackets bonded with nano-filled composites was assessed by Chalipa et al. [15]. Three composites were used in that study to bind the brackets together. Transbond XT was used in group 1 as a traditional bond. In the second and third groups, two nanocomposites, Filtek Supreme XT and Aelite esthetic enamel, were used, respectively. With respect to MPa, the shear bond stress values in the first, second, and third composites were all equal. Consequently, compared to conventional composites, a higher shear stress bond can be achieved by using particular types of nanocomposites.

Degrazia et al. [16] looked into the characteristics of an adhesive used in orthodontics that had halloysite nanotubes (TCN-HNT) loaded with triclosan. A control group consisted of a resin blend that had varying mass concentrations of the aforementioned nanostructures added to it, specifically 5%, 10%, and 20%. It was found that the addition of TCN-HNT increased the specifications for polymerization without changing the bonding properties right away. Furthermore, it was determined that adhesives containing nanostructures at concentrations greater than 10% might enhance long-term antimicrobial efficacy.

Nanoparticle Delivery from Elastomeric Ligature

The incorporation of silver nanoparticles into elastomeric ligatures, which are used in orthodontics to tie brackets and archwires, has been shown in studies to reduce dental biofilm and the demineralization of enamel caused by bacterial plaque accumulation. This alteration does not affect the material's mechanical or chemical properties, and as a result, it improves the efficacy of fixed orthodontic therapy. This novel application of nanotechnology to dental materials heralds improved antimicrobial properties [17].

Anti-cariogenic, anti-inflammatory, and antibiotic drug molecules embedded in an elastomeric matrix can be delivered via nanoparticles using elastomeric ligatures as a carrier scaffold. Previous reports in the literature describe the release of anti-cariogenic fluoride from elastomeric ligatures [16-18]. According to these studies, fluoride release is characterized by a logarithmic decrease after an initial burst of fluoride during the first few days. Monthly replacement of the fluoride ties is recommended for maximum clinical benefit [19,20].

Nanoimprinting of Power Chains

Another more recent idea, nanoimprinting, has enhanced the morphological and inherent qualities of power chains, which are used in orthodontics to close spaces between teeth. Power chains with nanoimprinting have nanostructures on their surface known as nanopillars. This technology solves the drawbacks of orthodontic auxiliaries by converting hydrophilic material to hydrophobic material [21].

Nanoparticles as Antimicrobial Agents

The demineralization of the enamel surfaces surrounding brackets or bands causes white spot lesions, an undesired side effect of orthodontic treatment. Caries and gingival inflammation are primarily caused by the patient's poor oral hygiene, which leads to bacterial accumulation and demineralization of the tooth surfaces.

Numerous studies have been conducted in the past to determine the best approaches for treating and preventing these lesions. In dentistry, the use of nanoparticles as antimicrobial agents is becoming more common. They can interact with the microbial membrane more closely and offer a larger surface area for antimicrobial activity because of their surface-to-volume ratio. There has been a surge in research on nanoparticles and their potential use as antimicrobials due to their distinct physiochemical properties and antimicrobial effects [22].

The antimicrobial properties of nanoparticles in biomaterials can be applied to orthodontics through two main mechanisms: either as coatings on surfaces or by mixing them with materials like glass ionomers or composites [21,22].

A variety of nanoparticles, including chitosan, zinc oxide, silver, titanium oxide, and copper oxide, have been shown to have antimicrobial properties [23,24].

Chitosan: As an antimicrobial agent, a hydrophilic biopolymer obtained mechanically through N-deacetylation of chitin can be utilized. In their study, Mirhashemi et al. [25] showed that adding chitosan to a composite at a concentration of 10% composite decreased the formation of biofilms when combined with zinc oxide.

Silver nanoparticles: Silver has been used for centuries to treat burns and wounds because of its known antibacterial properties. The adhesion of streptococci to orthodontic adhesives has been significantly reduced when silver nanoparticles have been added to the composites [14].

Additionally, silver was added to the Transbond XT primer at three different concentrations: 1%, 5%, and 10%. It was discovered that the addition of silver produced the desired effects at the concentrations of 1% and 5%, while increasing the concentration to 10% resulted in impaired physical properties [26].

Orthodontic brackets coated with nanosilver have been observed to reduce plaque adherence, which in turn lessens demineralization and the incidence of white spot lesions [27].

Copper: Orthodontic adhesives have also been enhanced with copper oxide nanoparticles. According to Toodehzaeim et al.'s research [28], adding copper oxide nanoparticles to Transbond XT at weight concentrations of 0.01%, 0.55%, and 1% inhibited the growth of *S. mutans*. Moreover, it was suggested that increasing the concentration enhanced the antimicrobial effect.

Nanocoated Orthodontic Mini-Screws/Temporary Anchorage Devices

Studies have evaluated the osseointegration, stability, and surface properties of nanoengineered mini-screws. Titanium dioxide, or TiO2, nanotubes are investigated using mini-screws that were compared to conventional mini-screws and laden with ibuprofen and recombinant human bone morphogenetic protein-2 (RhBMP-2). On human tissues, the effects of drug modification with nanoparticles and tissue health were positive. When combined with modified drugs such as aspirin, vitamin C, and antibiotic agents, these nanoparticle-modified mini-screws can help lessen human tissue inflammation at the location of mini-screw implantation in orthodontics. This modification has demonstrated improved surface and wettability characteristics of nanocoated mini-screws when compared to conventional mini-screws [27-30].

Nanosilver Mouth Rinse

The majority of mouthwashes typically contain ethanol, which some patients with periodontal infections find irritating because it inhibits the growth of bacteria. Using extremely low concentrations, silver nanoparticles were mixed with an ethanol-free mouthwash and compared with more affordable mouthwashes. They noticed that there was no difference between the commercially available mouth rinses and the silver nanoparticle built-up mouth rinses [31].

Enamel Remineralizing Agents

As a breakthrough in nanotechnology, nano-hydroxyapatite has been added to products for enamel remineralization. Recently, a paste consisting of calcium nanophosphate arranged in the crystalline form of hydroxyapatite was created. Because the surface area and wettability of hydroxyapatite nanoparticles are increased, calcium nanophosphate crystals smaller than 100 nm improve the bioactivity of the product. The surface of the demineralized tooth releases calcium, phosphate, and fluoride ions and organizes them into fluorapatite and CaF2. According to Medeiros et al.'s study [32], calcium nanophosphate shields the enamel surface from erosion by forming a protective layer on it.

Nanoparticle Acrylic Baseplates of Orthodontic Removable Appliances

Orthodontic stomatitis may not develop if *Candida albicans* proliferation under removable acrylic appliances is kept under control. The study of acrylic materials with nanoparticle inclusions having antimicrobial capabilities and their application in removable appliances is still in its initial stages and is restricted to in vitro models.

Silver nanoparticles were introduced to Selecta Plus and Rapid Repair, two varieties of acrylic resins, in a study conducted by Sodagar et al. [33]. The used acrylic liquids had nanoparticle concentrations of 0.2% and 0.05%. According to the observations, the flexural strength of Rapid Repair decreased when 0.05% of the nanoparticles were added, but it increased and nearly reached its initial value at 0.2% concentration. On the other hand, Selecta Plus's flexural strength increased when nanoparticles were added; however, the increase was more pronounced when 0.05% was added. It was determined that a few variables, including the kind of acrylic and the concentration of the nanoparticles, affected changes in flexural strength.

Nanocoated Clear Aligners

**Figure 4:**
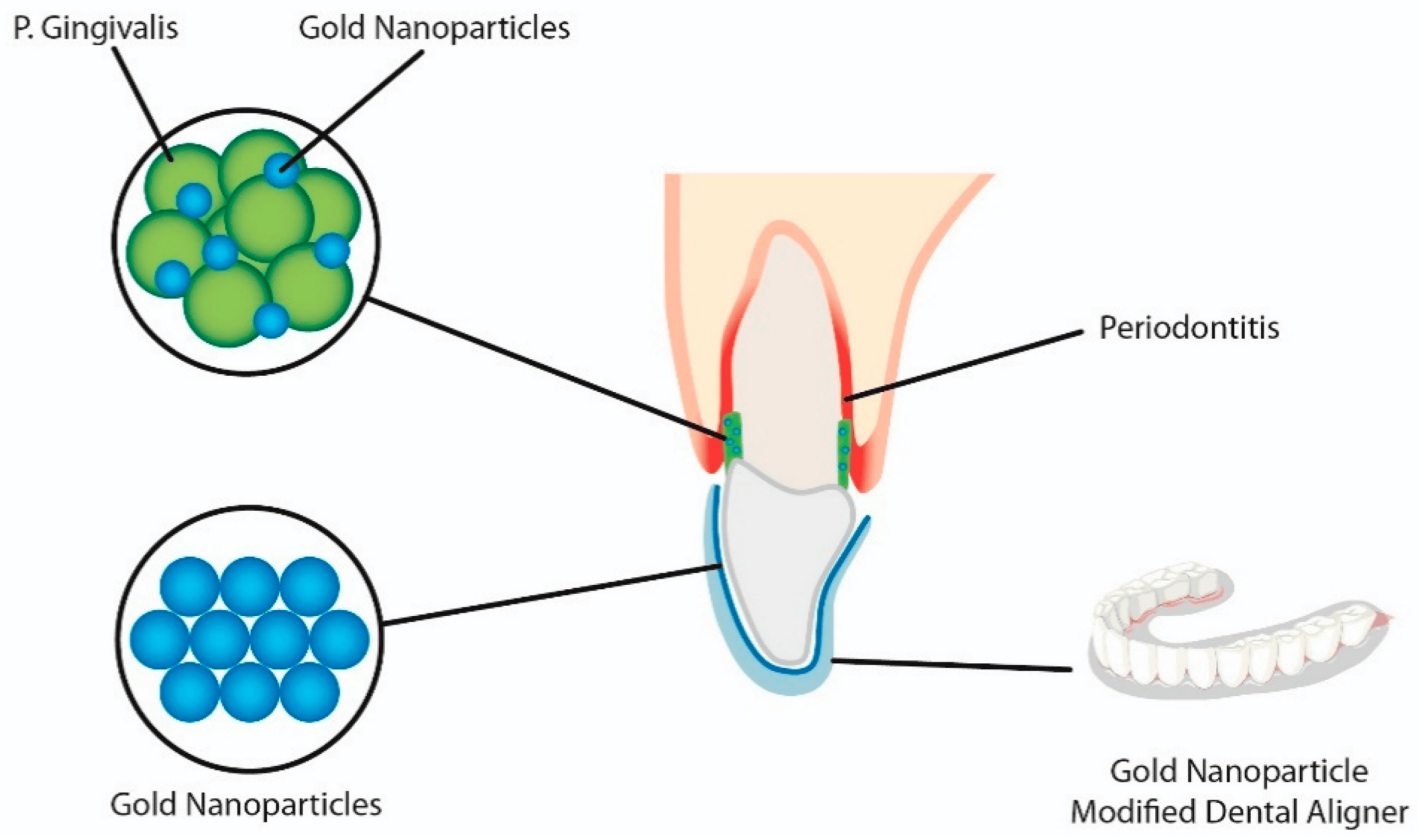
Nanocoated clear aligners Reference: [12]

As shown in Figure 4, orthodontic appliances like clear aligners can benefit from the application of nanosized gold particles and zinc oxide nanoparticles, which can enhance their antibacterial activity by inhibiting the formation of biofilm. Plaque accumulation is increased by the fact that aligners cover the teeth and gingiva for nearly the whole day. Zinc oxide nanoparticles [34] are effective against *S. mutans* for up to two days and up to seven days in vitro, while nanosized gold particles [35] are effective against *Porphyromonas gingivalis*. Positive biocompatibility is also demonstrated by gold particles in vitro and in vivo.

Future scope of nanotechnology in orthodontics

Shape-Memory Polymers

The field of adult orthodontics has grown, and with it, interest in using shape-memory polymers to create esthetic/tooth-colored orthodontic archwires has led to the introduction of a class of stimuli-responsive materials. They are able to retain their pre-programmed shape and can be stimulated by light, heat, or a magnetic field to revert to an original or desired shape [34,35]. Once in the mouth, these polymers can cause the movement of teeth through photoactive nanoparticles activated by light or body temperature. Shape-memory polymers also have the qualities of clarity, stain resistance, and esthetic appeal [36,37]. With fewer patient visits, the application of continuous forces over a wide range of tooth movement is made possible by the shape-memory polymer appliance's high percentage elongation (up to 300%) [38].

Biological Microelectromechanical Systems (Bio-MEMS)/Biological Nanoelectromechanical Systems (Bio-NEMS)

The science and technology working at the microscale for biological and biomedical applications, where the electronic and mechanical functions can be included, is known as bio-MEMS. Conversely, bio-NEMS are apparatuses that combine mechanical and electrical functions at the nanoscale [39]. In the clinical domain, the two primary application areas are therapeutics (drug delivery microchips) and diagnostics (using implantable sensors). Nanoscale devices that combine mechanical and electrical functionality are called nanoelectromechanical frameworks or NEMS.

It has been suggested that orthodontic tooth movement can be accelerated by adding electricity to these mechanical forces [39,40]. Enzymatic micro-batteries have the potential to function as an electrical force hotspot to accelerate orthodontic tooth movement when applied to the gingiva in close proximity to the alveolar bone [41-42]. This non-osseointegrated device can run on glucose as fuel. However, a few issues still need to be resolved, like soft tissue biocompatibility and the impact of different foods on the pH and temperature of a microfabricated protein battery. It is very likely that orthodontic tooth movement will be improved in the coming years by using MEMS-/NEMS-based systems.

Nano-Low-Intensity Pulsed Ultrasound (LIPUS) Devices

Devices that use LIPUS transmit mechanical energy in the form of acoustic pressure waves that are above the human hearing limit and pass through and into biological tissues [43]. LIPUS has demonstrated efficacy in facilitating wound healing, augmenting bone growth, facilitating bone healing following fractures [44], decreasing root resorption, and enhancing tooth movement.

It has been demonstrated to be successful in both human and animal mandibular growth stimulation [43,44]. The effect of LIPUS on tooth movement and root resorption in orthodontic patients was assessed in a recent randomized controlled trial by El-Bialy et al. [45-47]. It was discovered to be useful in both minimizing root resorption and speeding up tooth movement. Patients utilizing Invisalign clear aligners experienced a statistically significant reduction in the length of their orthodontic treatment, according to another retrospective clinical trial conducted by Kaur et al. [48]. As a result, LIPUS can be viewed as a potentially helpful tool in orthodontics, and more research is needed to fully comprehend its uses in this field.

Smart Brackets With Nanomechanical Sensors

An integrated sensor system for 3D force and moment measurement is part of an intelligent bracket concept that was recently published. Nanomechanical sensors can be made to be incorporated into the base of orthodontic brackets to provide the orthodontist with instantaneous feedback regarding the applied orthodontic forces. With the least amount of negative effects, the orthodontist will be able to move teeth efficiently by adjusting the applied force to be within a biological range.

The main advantages of smart brackets are the ability to move teeth more effectively, manage teeth precisely in all directions, spend less time at the chairside, and reduce treatment time [49-51].

Nanotechnology and Gene Therapy in Orthodontics

It can be challenging to regulate the intricate interaction between inherited and environmental factors that can lead to mandibular underdevelopment. It has been demonstrated that bite-jumping appliances can promote development. However, some research has cast doubt on these functional appliances' long-term clinical value in promoting mandibular growth.

Similar to other medical specialties, nanotechnology is becoming more significant in relation to mandibular growth. It has been mentioned that certain vascular growth-inducing genes play a role in mandibular growth [52,53]. In order to ascertain the safety and effectiveness of gene therapy in orthodontics, additional clinical trials are necessary.

Nanorobots in Orthodontics

There are several uses for nanorobots in the medical sciences. These tools can be used for local anesthetic, hypersensitivity treatment, and other purposes. Apart from the aforementioned uses, orthodontic procedures can also employ nanorobots.

By employing nanorobots, orthodontic treatment times can be shortened. In just a few minutes, orthodontic robots can painlessly rotate and vertically reposition teeth, whereas traditional methods take hours to complete [54,55]. Furthermore, nanorobots have the ability to directly manipulate periodontal tissues [54-57], which qualifies them as useful instruments for use in orthodontic therapy.

Nanorobotic dentifrice: Dentifrobots are a type of nanorobot that are incorporated into mouthwash or toothpaste to keep the tooth surfaces surrounding brackets free of bacteria and plaque. These nanorobots work to prevent the buildup of plaque and biofilm by breaking down trapped calculus or organic matter into inorganic or harmless substances, thereby continuously removing plaque and calculus. Additionally, these can act as a barrier against the bacterial putrefaction that causes an unpleasant oral odor [58,59].

Current concerns of nanotechnology in orthodontics

Although microorganisms and cancer cells benefit from nanoparticles, normal cells are also harmed by them. This is primarily due to oxidation and damage to cell membranes, DNA, and mitochondria, which can disrupt intracellular transport, cell division, and mutagenesis. Therefore, they may result in apoptosis, necrosis, inflammation, and reactive oxygen species (ROS). When employed in the oral cavity, nanoparticles have the ability to be absorbed, with their small size facilitating their easy penetration of biological systems and their rapid delivery to all regions of the body, including the brain. The toxicological profile of nanoparticles is influenced by various factors, including their dosage, distribution, metabolism, and disposal.

The majority of orthodontics research is done in vitro, with little investigation into toxicity, and the application of orthodontics in in vivo studies has been fairly tentative [60,61]. Prior to their routine application in patient care, more data and research are likely required. According to Asmatulu et al. [62], nanotechnology has some unfavorable effects on the environment that could harm ecosystems and human health currently.

## Conclusions

Owing to its wide range of applications, nanotechnology holds great promise for the field of orthodontics, even though its use is still in its initial stages. The potential risks of nanotechnology are a source of concern due to the scarcity of data and ignorance about their detrimental effects, which makes it difficult to regulate the safety of nano-therapeutics.

Overall, nanotechnology will be a significant factor in orthodontic therapy going forward if all current efforts in clinical application are successful and come at a reasonable cost to the orthodontist and patients.
